# Genetic diversity in populations of *Isatis glauca* Aucher ex Boiss. ssp. from Central Anatolia in Turkey, as revealed by AFLP analysis

**DOI:** 10.1186/1999-3110-54-48

**Published:** 2013-11-04

**Authors:** Özlem Özbek, Elçin Görgülü, Şinasi Yıldırımlı

**Affiliations:** 1 Department of Biology, Faculty of Art and Science, Hitit University, Ulukavak Mah. Çiftlik Çayırı Cd. No: 61, Çorum, Turkey; 2 Department of Biology, Faculty of Science, Hacettepe University, Ankara, Turkey

**Keywords:** AFLP, Genetic diversity, *Isatis glauca* subspecies, ssp. *galatica*, ssp. *glauca*, ssp. *sivasica*

## Abstract

**Background:**

*Isatidae* L. is a complex and systematically difficult genus in *Brassicaceae*. The genus displays great morphological polymorphism, which makes the classification of species and subspecies difficult as it is observed in *Isatis glauca* Aucher ex Boiss. The aim of this study is characterization of the genetic diversity in subspecies of *Isatis glauca* Aucher ex Boiss. distributed widely in Central Anatolia, in Turkey by using Amplified Fragment Length Polymorphism (AFLP) technique.

**Results:**

Eight different *Eco* RI-*Mse* I primer combinations produced 805 AFLP loci, 793 (98.5%) of which were polymorphic in 67 accessions representing nine different populations. The data obtained by AFLP was computed with using GDA (Genetic Data Analysis) and STRUCTURE (version 2.3.3) software programs for population genetics. The mean proportion of the polymorphic locus (*P*), the mean number of alleles (*A*), the number of unique alleles (*U*) and the mean value of gene diversity (*He*) were 0.59, 1.59, 20, and 0.23 respectively. The coancestry coefficient (*ϴ*) was 0.24. The optimal number of *K* was identified as seven. The principal component analysis (PCA) explained 85.61% of the total genetic variation.

**Conclusion:**

*Isatis glauca* ssp. populations showed a high level of genetic diversity, and the AFLP analysis revealed that high polymorphism and differentiated subspecies could be used conveniently for population genetic studies. The principal coordinate analysis (PCoA) based on the dissimilarity matrix, the dendrogram drawn with UPGMA method and STRUCTURE cluster analysis distinguished the accessions successfully. The accessions formed distinctive population structures for populations AA, AB, E, K, and S. Populations AG1 and AG2 seemed to have similar genetic content, in addition, in both populations several hybrid individuals were observed. The accessions did not formed distinctive population structures for both populations AI and ANP. Consequently, Ankara province might be the area, where species *Isatis glauca* Aucher ex Boiss. originated.

**Electronic supplementary material:**

The online version of this article (doi:10.1186/1999-3110-54-48) contains supplementary material, which is available to authorized users.

## Background

**Figure 1:**
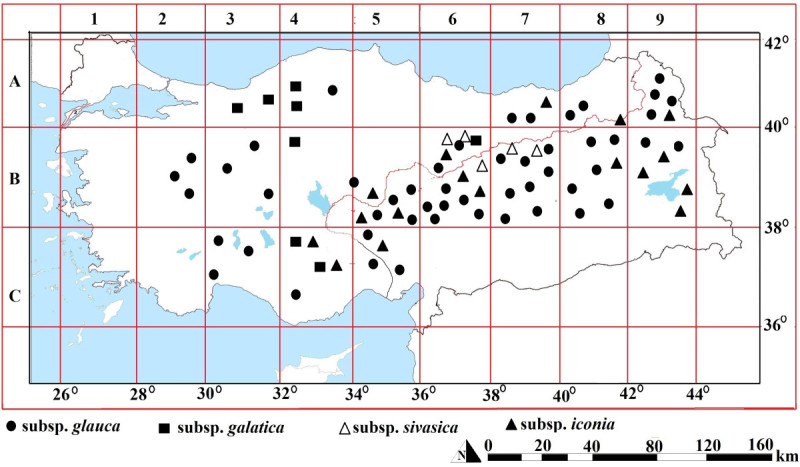
**Distribution of** ***Isatis glauca*** **Aucher ex Boiss. subspecies in Turkey.** The subspecies on the right side of the diogonal line were reported by Mısırdalı (1985) and the subspecies on the left side of the diogonal line were reported by Yıldırımlı (1988).

*Isatis glauca* Aucher ex Boiss., is a diploid biennial or perennial herbaceous species with a chromosome number set of 14 (2n = 28). *Isatis glauca* has four recognized subspecies, *glauca*, *galatica* Yıldırımlı, *sivasica* (Davis) Yıldırımlı and *iconia* (Boiss.) Davis. The distribution of this polymorphic species in Turkey is represented in Figure 1 (Yıldırımlı 1988).

Taxonomic and cytological investigations on the *Isatis* L., species grown in the Eastern and South Eastern Anatolia and across the Eastern Mediterranean region were made by Mısırdalı (1985) (Figure 1, the region on the left side of red diagonal line on the map of Turkey). Mısırdalı (1985) displayed that there were 31 species, 14 subspecies, and 2 varieties in *Isatis* collections from East & Southeast Anatolia, and the Eastern Mediterranean - regions in Turkey. Yıldırımlı and Doğan (1986) investigated palynological polymorphism in 790 pollen grains collected from *Isatis glauca* ssp. *glauca* flowers in Sivas province of Turkey. They observed that 505 (62.92%) of those pollen grains were tricolpate (normal), 129 (16.33%) were syncolpate, 87 (11.01%) were stefanocolpate and 69 (8.73%) were dicolpate. Gilbert et al. (2002) screened genetic diversity in *Isatis* ssp. (*Isatis tinctoria* L., dyer’s woad) by using AFLP. Al-Shehbaz et al. (2006) discussed the evaluation of characters and their utilisation in infrafamilial classifications, delimitations of genera, the collection of molecular data and major subdivisions of the family, problematic taxa and future challenges in the *Brassicaceae* (*Cruciferae*). Kızıl (2006) reported morphological and agronomical characteristics of some wild and cultivated *Isatis* species. Moazzeni and Zarre (2006) studied the presence of *Isatis tinctoria* in Iran by using its morphological characters. Moazzeni et al. (2007) examined the systematic application of seed-coat surfaces of 23 species (41 populations) in four genera of tribe *Isatidae* using scanning electron microscopy (SEM). Spataro et al. (2007) displayed genetic variation and population structure in a Eurasian collection of *Isatis tinctoria* L. by using AFLP and SAMPL molecular markers. Spataro and Negri (2008) analysed phenotypic and genetic diversity of a Eurasian collection of *Isatis tinctoria* as well as its adaptability according to a wide range of altitudes. Tu et al. (2008) assessed intertribal somatic hybrids between *Brassica rapa* and *Raphanus sativus* with dye and medicinal plant *Isatis indigotica*. Du et al. (2009) presented the production and cytogenetic characterisation of intertribal somatic hybrids between *Brassica napus* and *Isatis indigotica*, and backcrosses. Tu et al. (2009) showed the chromosome elimination, addition, and introgression in intertribal partial hybrids between *Brassica rapa* and *Isatis indigotica*. Moazzeni et al. (2010) analysed the phylogeny of 28 Iranian taxa of *Isatidae* by using ITS sequence of ribosomal DNA and morphological characters. Rocha et al. (2011) detected genetic diversity in woad (*Isatis tinctoria* L.) by using ISSR markers. All these studies reveal that although there are many reports on morphological and genetic variation in the *Brassicaceae* family, very few studies are found on morphological or genetic variation in *Isatis glauca* Aucher ex Boiss.

The aim of this study was to analyse genetic diversity and population structure using molecular marker techniques among the *I. glauca*, subspecies populations from Central Anatolia in Turkey. AFLP method was used to estimate genetic diversity (*He*), population structure and genetic differentiation between populations using coancestry coefficient *ϴ*_P_ (= *F*_ST_). In addition, the study also aimed to find the effects of climate (temperature *T*, humidity *HU*, and rainfall *R*), and geography (altitudes *AL*, latitudes *LA* and longitudes *LN*) on the genetic diversity of nine *I. glauca* populations.

## Materials and Methods

### Materials

**Figure 2:**
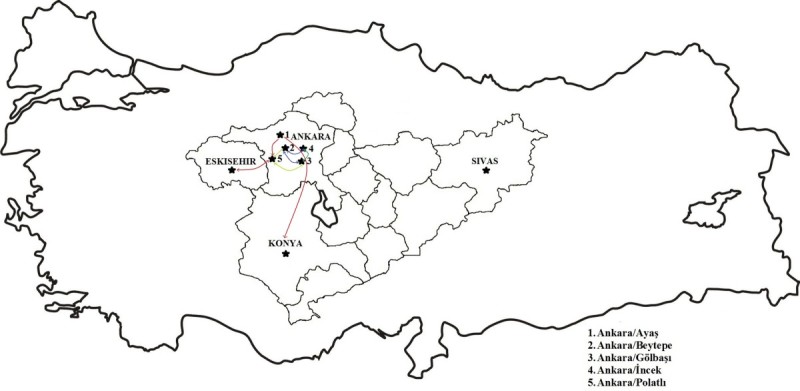
**Presentation of the locations, where nine** ***Isatis glauca*** **Aucher ex Boiss. subspecies populations were collected from Central Anatolia in Turkey.** The coloured lines represents the gene flow between populations according to STRUCTURE analysis results.

In this study, genetic diversity of 67 accessions from nine *I. glauca* subspecies populations collected from Ankara/Ayas (AA), Ankara/Beytepe (AB) valley, Ankara/Incek (AI), Ankara/Golbasi1 (AG1), Ankara/Golbasi2 (AG2), Ankara/Polatlı (ANP), Eskisehir (E), Konya (K) and Sivas (S) (Figure 2) in May and July of 2011. For amplified fragment length polymorphism (AFLP) analysis, one individual was used from each accession. The Additional file 1: Table S1 contains also population codes (PC), accession numbers (AN), and subspecies names. Throughout the text, the different populations were identified based on those codes. Plant specimens, are kept in Hacettepe University Herbarium (HUB) and identified by Prof. Dr. Şinasi Yıldırımlı.

### Methods

#### Fluorescent based AFLP analysis

Genomic DNA was extracted from 1 to 1.5 month old leaves of individual plants from each of 67 accession using CTAB method, as described by Kidwell and Osborn (1992). AFLP analysis was carried out as described by Vos et al. (1995), with minor modifications. The primers, adaptors, and altogether, eight primer combinations used in this study are given in Additional file 2: Table S2 and Additional file 3: Table S3 respectively.

#### Restriction-ligation reaction

The aliquots of the restriction-ligation master-mix (a-RLMM) were prepared in a 2 mL Eppendorf tube without enzymes and the T4 DNA ligase buffer 10x. In a- restriction-ligation master-mix (RLMM) for one sample, the following were added; 0.617 μL H2O, 1.1 μL NaCl (0.5 M), 0.55 μL BSA 10× (1 g/L), 1 μL *Mse* I adaptor (50 pmol/μL = 50 μM) and 1 μL *Eco* R I adaptor (5 pmol/L = 5 μM). The RLMM was prepared by using 1.1 μL aliquots T4 DNA ligase buffer 10x, 4.267 μL a-RLMM, 0.05 μL *Mse* I (20 U/μL) enzyme, 0.05 μL *Eco* R I (100 U/μL) enzyme, and 0.033 μL T4 DNA ligase enzyme (30 weissU/μL). 96 well PCR microtiter plates were used for 67 samples. For each sample a 5.5 μL RLMM aliquot was dispensed into the wells and the 5.5 μL of the diluted sample genomic DNA (50 ng) was added. PCR microtiter plates were short spinned in a centrifuge (Sigma 1–14) and left for incubation (Techne TC-412) at 37°C for 6 hours.

#### Preselective PCR amplification

Before the preselective PCR amplification, the 5-μL restriction-ligation products were diluted by adding 95 μL TE (1X). For the preselective PCR amplification, the primers, were complementary to the sequences of the *Eco* R I and *Mse* 1 adaptors, that contained one selective nucleotide. Preselective PCR, reaction consisted of 2 μL (10X) PCR buffer, 1.6 μL (2 mM) MgCl_2,_ 1.2 μL (2.5 mM) dNTPs, 1 μL (5 pmol) *Eco* R I primer, 1 μL (5 pmol) *Mse* I primer, 0.2 μL (1 μ/μL) *Taq* DNA polymerase, 9 μL ddH_2_O, and 4 μL diluted restriction-ligation products. The diluted restriction-ligation products were kept at −20°C until used. Preselective amplification for 20 cycles was carried out through PCR. Initial extended denaturation was carried out at 94°C for 2 min, followed by denaturation at 94°C for 30 sec, annealing at 56°C for 30 sec, extension at 72°C for 2 min, and the final extension at 60°C for 30 min.

#### Selective PCR amplification

Selective PCR amplification was carried out using 10 μL preselective PCR amplification products diluted in 190 μL TE (1X). The diluted preselective PCR amplification products were kept at −20°C until used. The selective PCR amplification was done with *Eco* R I and *Mse* I primers, which contained three selective nucleotides. For the fluorescent-based fragment analysis, the *Eco* R I selective primers of 39, 36, 39, and 36 were labelled with FAM-1, FAM-2, FAM-3, and FAM-4 respectively, while the *Eco* R I selective primers 33, 36, 33, and 36 were labelled with VIC-1, VIC-2, VIC-3, and VIC-4 fluorescent dye. *Mse* I primers were used with the unlabelled ones. The selective PCR amplification used in this study is given in Additional file 4: Table S4.

#### Capillary electrophoresis

Capillary electrophoresis of selective amplification products were performed by using ABI 3130 × l (Applied Biosytems Inc., Foster City, CA). For capillary electrophoresis; 1 μL selective PCR amplification products were denatured using 0.2 μL LIZ-500 size standard at 95°C for 5 min followed by heat shock applied by chilling on ice at 0°C for 5 min. Then, permanently denatured single stranded DNA was loaded on ABI 3130 X l. After capillary electrophoresis, AFLP fragments were scored into a binary data matrix as 1 (present) or 0 (absent) using Gene Mapper 4.0 software package (Applied Biosystems).

#### Statistical analysis

For investigation of population structures of nine *I. glauca* ssp. populations, we used the software program STRUCTURE (Pritchard et al. 2000). STRUCTURE provides a model-based Bayesian approach to explain population structure by using our entire AFLP markers data set to identify *K* clusters to which the program then assigns each individual. We used 67 individuals to infer the optimal value of *K* (ie., the number of clusters) by evaluating *K* = 1–9. For parameter set, in our model, we selected with admixture as ancestry model and the allele frequencies were assumed to be correlated, since it is more reasonable to assume common ancestry of such closely related populations. The length of burn-in Markov Chain Monte Carlo (MCMC) replications was set to 10000 and data were collected over 100000 MCMC replications in run, based on previous literature suggesting that this level is sufficient (Evanno et al. 2005). We determined the optimal value of *K* using the second order statistics (∆K) developed by Evanno et al. (2005) and the ad hoc procedure described by Pritchard et al. (2000).

**Figure 3:**
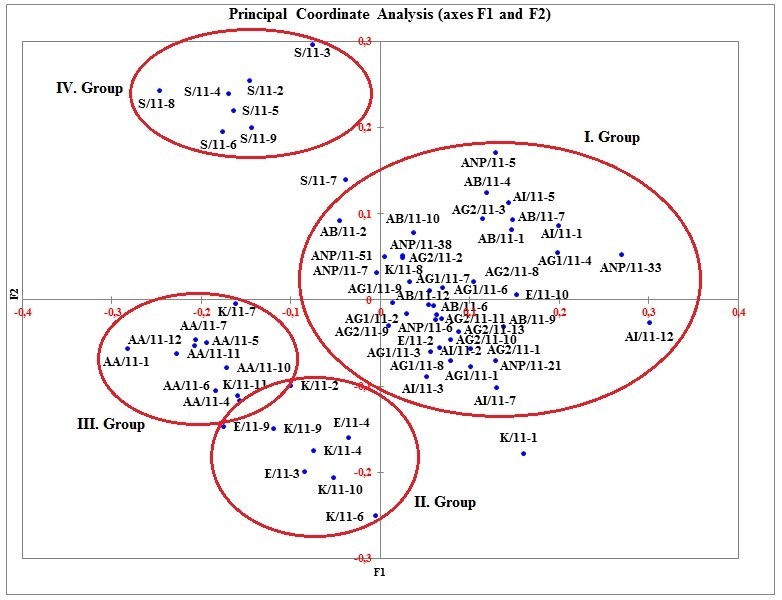
**The scattered plot of the first and second principal coordinates obtained from eight AFLP primer combinations in 67 accessions from nine** ***Isatis glauca*** **Aucher ex Boiss. subspecies populations.**

**Figure 4:**
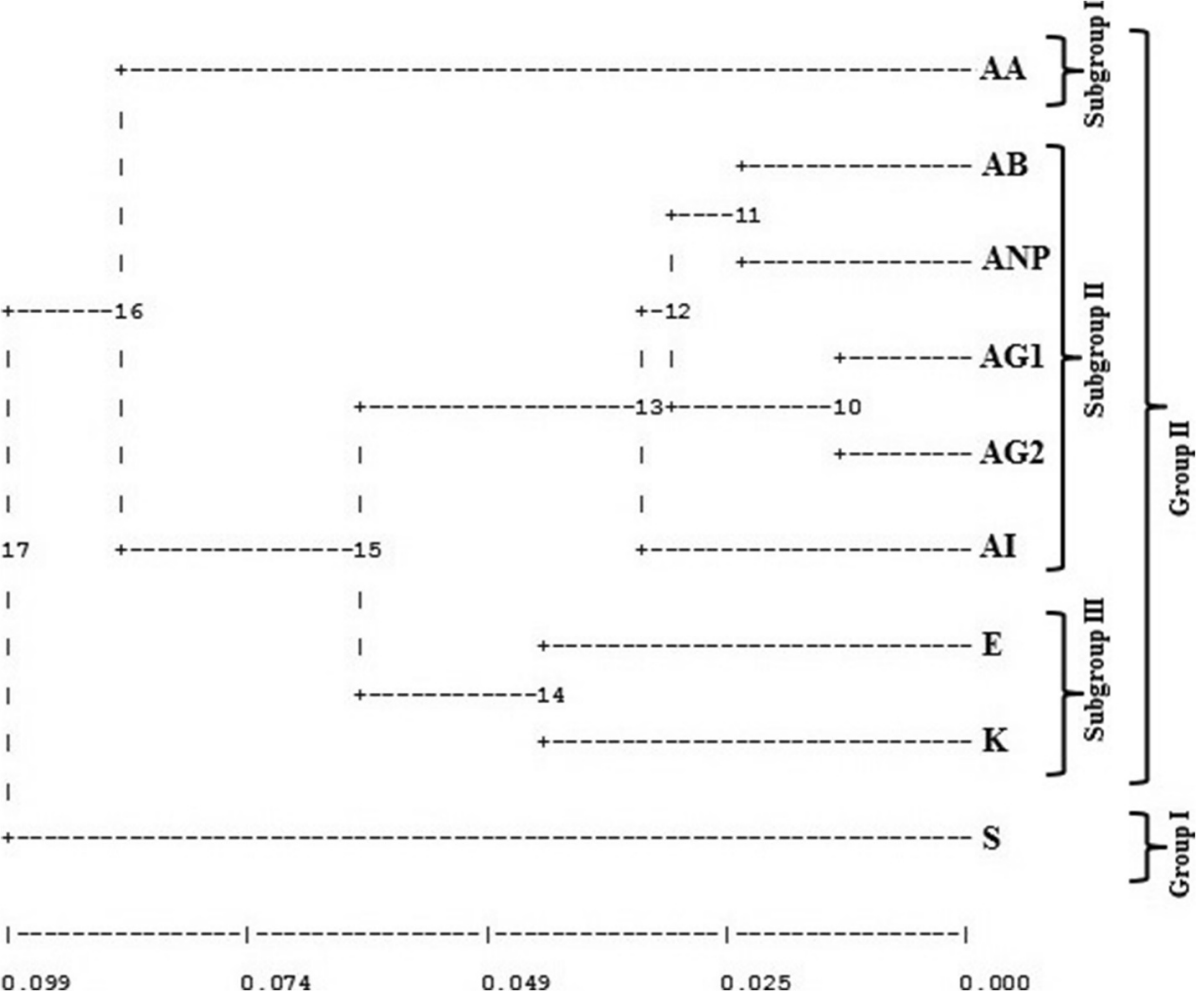
**A dendrogram, which was constructed according Nei’s (** 1978 **) genetic distance and UPGMA method displaying the relatedness between nine** ***Isatis glauca*** **Aucher ex Boiss. subspecies populations collected from Central Anatolia in Turkey.**

To confirm the results obtained from STRUCTURE, we conducted two additional analyses enabling us to visualise distribution of individuals. First, we performed Principal Coordinate Analysis (PCoA). PCoA is a scaling or ordination method that starts with a matrix of similarities or dissimilarities between a set of individuals. The method aims to produce a low-dimensional graphical plot of the data in such a way that distances between the points in the plot are close to the original dissimilarities. For PCoA, the dissimilarity matrix values were used to ordinate 67 accessions representing nine Turkish *Isatis glauca* subspecies populations on a scattered plot using XLSTAT (version 2013, Addinsoft™), a software package for Windows (Figure 3). Second, a dendrogram (Figure 4) was constructed according to Nei’s (1978) genetic distance and UPGMA method using the software program GDA (Genetic Data Analysis; Lewis and Zaykin 2001) version 1.1., which is a software package for analysis of discrete population genetics data according to Weir (1996).

### Principal component analysis (PCA)

PCA was calculated by using the following genetic indices, and the sample sizes: *P*, *A* and *He*, and *N* respectively, as well as geographical (*AL*, *LA*, and *LN*) and climatic (*RA*, *T*, and *HU*) data (Additional file 5: Table S5) as variables according to the Pearson’s (one-tailed) correlation matrix using XLSTAT (version 2013, Addinsoft™) a software package for Windows.

Since AFLPs are dominant markers, data were scored as binary data. Polymorphic bands, exhibiting presence or absence of bands, were scored as alternative alleles. The data thus obtained were considered as haploid and analysed by GDA.

Gene diversity () (Nei *He*^1973^), estimating within-population diversity, was computed as the expected heterozygosity based on allele frequencies for each locus and for all loci, polymorphic and non-polymorphic ones.

Genetic differentiation between populations is often estimated with the Nei’s coefficient *G*_ST_ (Nei 1973) for dominantly inherited DNA markers. This coefficient may tell us how genetic variation is partitioned within- and between-populations; a high *G*_ST_ value indicates that plants within a population are relatively similar, but populations are considerably different. When there are two alleles (1 and 0), this *G*_ST_ is identical to Wright’s (1951) *F*_ST_ (Nei 1973). Thus, *F* statistics was used to estimate the extent of differentiation, between groups (populations and sub-populations) (Hartl and Clark 1997). The coancestry coefficient *ϴ* (= *F*_ST_) was measured for every locus and for all loci in each population.

Multiple regression analysis (MR) (SPSS version 11 for Windows) was conducted with AFLP diversity and allele diversity in nine populations. MR employed the genetic variables (*P*, *A* and *He*) as dependent and the environmental variables (*AL*, *LA*, *LN*, *RA*, *T*, and *HU*) as independent.

#### Reproducibility of AFLP profiles

The reproducibility of scoring of the AFLP profiles was checked by analysing the genotyping error rate in repeated runs of the same extracts from eight plants. In the beginning of the study, 12 different primer combinations were scanned for eight samples (accessions) and the eight polymorphic primer sets selected and applied for all of the 67 accessions. The data were obtained using selected eight primer combinations in both pre-scanning and applied through all accessions including those eight accessions were compared to evaluate the reproducibility of AFLP markers. The percent of reproducibility was calculated for each primer combination. Average percent of reproducibility was calculated as 90.62% that ranged 86.38% (E36XM35) to 97.78% (E36XM34) among the primer combinations.

## Results

### AFLP fragment analysis

In AFLP analysis, eight *Eco* R I and *Mse* I primer combinations generated 805 fragments. The primer combination E36xM34 produced the highest number (194) of fragments, while the primer combination E36xM33 produced the least number (32) of fragments (data not shown). The primer combination E36xM34 displayed the highest number (510) of fragments in population AA in total of eight individuals, while the primer combination E39xM41 displayed the least number (81) of fragments in population AI in total of six individuals. Average number of fragments per population was calculated according to total present fragment numbers produced by eight primer combinations in all individuals of each population and ranged 252.17 (in population AI) to 314.75 (in population AA).

### Genetic analysis

In AFLP analysis, eight *Eco* R I and *Mse* I primer combinations produced 805 fragments (loci), 793 (98.5%) of which were polymorphic and 12 (1.5%) were monomorphic. The proportion of the mean number of the polymorphic loci (*P* = 0.59), the mean number of alleles (*A* = 1.59) and the mean value of the genetic diversity (*He* = 0.23) for nine populations, are represented in Table 1. Population K has the highest proportion of polymorphic loci (*P* = 0.68), the highest number of alleles (*A* = 1.68), and the highest value (*He* = 0.26) of genetic diversity (Table 1). Population AI has the lowest proportion of polymorphic loci (*P* = 0.50) and the lowest number of alleles (*A* = 1.5), while population S has the lowest genetic diversity value (*He* = 0.20).

**Table 1:** **The number of sample size (** ***N*** **), the proportion of polymorphic locus (** ***P*** **), number of alleles (** ***A*** **), the genetic diversity value (** ***He*** **) and unique allele (** ***U*** **) numbers per population, and coancestry coefficient (** ***ϴ*** **) for studied nine Turkish** ***Isatis glauca*** **subspecies populations**

POP	***N***	***P***	***A***	***He***	***U***	***ϴ***
AA	8	0.60	1.60	0.22	2	
AB	8	0.60	1.60	0.22	-	
AG1	8	0.58	1.58	0.21	1	
AG2	8	0.61	1.61	0.23	3	
AI	6	0.50	1.50	0.22	-	
ANP	7	0.60	1.60	0.23	-	
E	5	0.56	1.56	0.25	1	
K	9	0.68	1.68	0.26	-	
S	8	0.54	1.54	0.20	12	
Mean		0.59	1.59	0.23		0.24

The mean value of the coancestry coefficient *ϴ* (= *F*_ST_) or genetic differentiation between the populations is 0.24 (95% confidence interval) (Table 1). The highest genetic distance (*GD* = 0.24) was observed between population AA and population S, while the lowest genetic distance (*GD* = 0.04) was observed between AG1 and AG2 (Data not shown).

### Unique alleles

In AFLP analysis of nine *I. glauca* ssp. populations, 19 unique alleles were observed (Additional file 6: Table S6). About the unique alleles, two existed in population AA, 1 in each of populations AG1 and E, 3 in population AG2 and 12 in population S. The frequencies of unique alleles range 0.13 (in populations AG1, AG2 and S) to 1.00 (population S).

### Population structure

**Figure 5:**
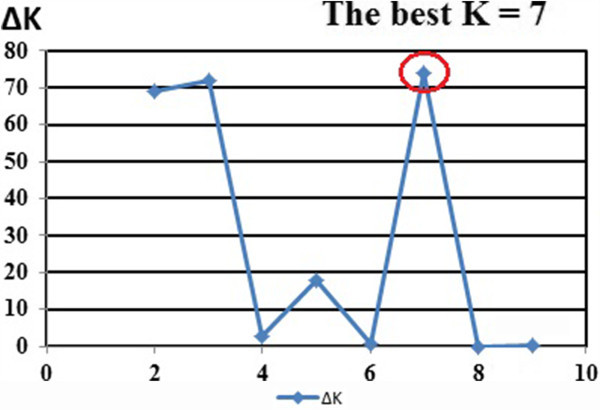
**The second order statistics (∆K) developed by Evanno (2005) for STRUCTURE in order to determine the number of subpopulations identified the optimal value for** ***K.***

**Figure 6:**
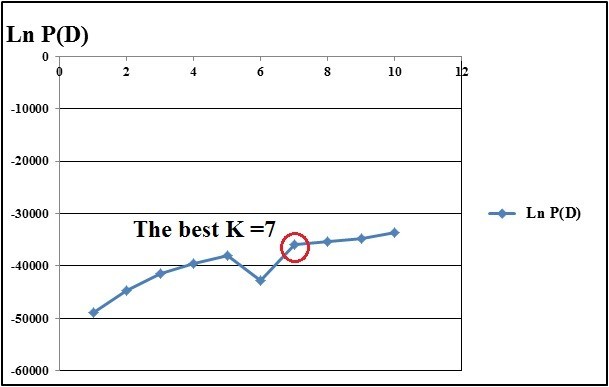
**The ad hoc procedure described by Pritchard et al. (** 2000 **) to determine the number of subpopulations identified the optimal value for** ***K.***

**Figure 7:**
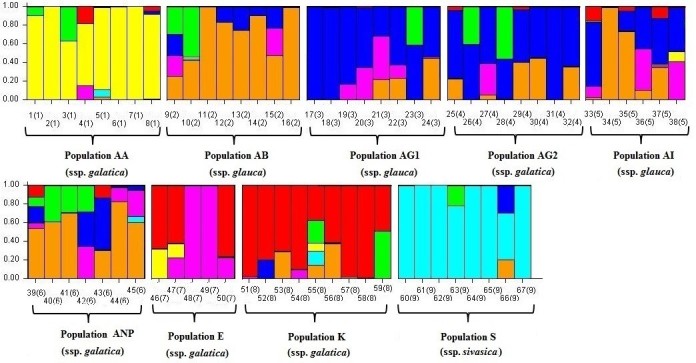
***Isatis glauca*** **Aucher ex Boiss. subspecies population structure based on Bayesian inference among 67 accessions analysed with 8 AFLP primer combinations assuming K = 7.**

The second order statistics developed by Evanno (2005) for STRUCTURE in order to determine the number of subpopulations identified the optimal value for *K* = 7 (Figure 5). It was confirmed by also the ad hoc procedure (Figure 6) developed by Pritchard et al. (2000). This means that the set of accessions was partitioned into seven clusters, which corresponded to the ssp. *galatica*, ssp. *glauca* and ssp. *sivasica*. When the coloured individual bar plot (Figure 7) was examined, some accessions had varying proportions of their genome from other clusters.

Accessions formed distinctive population structures for populations AA, AB, E, K, and S. Populations AG1 and AG2 seemed to have similar genetic content, in addition in both populations several hybrid individuals were observed. Populations AI and ANP did not display distinctive population structures. In these populations, all individuals, except one individual in population AI, were seemed to be hybrids. In general, all populations have hybrid individuals.

The hybrid individuals detected in this study is an indication of gene flow between populations. Particularly, extensive gene flow was observed between AG1, AG2, AI, and ANP. It is noteworthy that there is a population (light green colour) found in hybrid individuals, although there is no such a distinctive population among the populations analysed in this study. This might be because of there is no any sample from this population among the analysed populations or that might be extinct in the evolutionary history of subspecies populations. Therefore, some individuals are still carrying on the genetic material on their genetic background.

### Cluster analysis (UPGMA)

In the dendrogram, there are two main groups (Figure 4). All the populations except, population S clustered together in the first main group, while population S (*I. glauca* ssp. *sivasica*) clustered in the second main group. In the first main group, population AA (*I. glauca* ssp. *galatica*) separated clearly from rest of the populations in the first subgroup. In the second subgroup populations clustered into further subgroups. The populations AG1 (*I. glauca* ssp. *glauca*) and AG2 (*I. glauca* ssp. *galatica*), populations AB (*I. glauca* ssp. *glauca*) and ANP (*Isatis glauca* ssp. *galatica*), and population AI (*I. glauca* ssp. *glauca*) clustered together in the second subgroup. In third subgroup, population K (*Isatis glauca* ssp. *galatica*) and E (*Isatis glauca* ssp. *galatica*) clustered together.

### Principal components analysis (PCA)

The three extracted principal components according to PCA, explained 85.62% of the genetic variation (Table 2). The first component, which accounted for 37.06% of the total variance, was formed by *P*, *A*, *He*, and *AL* (Additional file 7: Table S7). The second component, representing 29.49% of the total variance, was formed by *N*, *LN*, and *RA*, while the third component represented 19.06% of the total variance, which composed of *LA, T*, and *HU*.

**Table 2:** **Total variance explained by principal component analysis (PCA) was performed by using data of sample size** ***N*** **, genetic indices (** ***P*** **,** ***A*** **, and** ***He*** **), climatic (** ***T*** **,** ***RA*** **, and** ***HU*** **), and geographical (** ***AL*** **,** ***LA*** **, and** ***LN*** **) as variables according to Pearson’s correlation (one-tailed) matrix with Eigen values, percentage of variance and cumulative percentage of variance**

Component	Eigen value	Variance (%)	Cumulative variance (%)
1	3.72	37.15	37.15
2	2.95	29.45	66.59
3	1.90	19.03	85.62

### Principal coordinates analysis (PCoA)

The three coordinates, which obtained according to the PCoA, explained 29.23% of total variation. The first axis (Eigen vector) displayed 11.96% of the variance, while the second axis (Eigen vector) and the third axis (Eigen vector) showed 9.41% and 7.85% of the variance respectively (data not shown). The codes of 67 accessions are plotted according to the first and the second axis that corresponded to different regions, from where nine *I. glauca* subspecies populations collected (Figure 3). The accessions from populations AA, E, K and S seperated clearly, the accessions from populations AB, AG1, AG2, AI and ANP were clustered in the same region.

### Multiple regression analysis

We employed the multiple regressions (MR) to determine AFLP allelic diversity. The results of MR analysis showed that the combination of altitude, latitude, longitude, temperature, humidity, and rainfall accounted for a considerable proportion of the variation observed in AFLP fragments diversity (Table 3). When combined, these six variables had effects on the variances of the mean proportion of polymorphic locus (92.8%), the mean frequency of alleles (74.9%), and the mean genetic diversity (74.9%); Overall, MR results indicated that climatic and geographic variables in combination with each other affected genetic diversity at a considerable level in Turkish Isatis *glauca* ssp. populations.

**Table 3:** **Displaying the effects of eco-geographical factors on genetic indices by multiple regression analysis (Abbreviations: Dependent variable DV, independent variable IV, coefficient of multiple regression R** ^**2**^ **, proportion of polymorphic locus** ***P*** **, the mean number of allele** ***A*** **, the mean value of genetic diversity** ***He*** **, temperature** ***T*** **, rainfall** ***RA*** **, humidity** ***HU*** **, altitude** ***AL*** **, latitude** ***LA*** **and longitude** ***LN*** **)**

DV	R^2^	IV
*P*	0.928	*T, RA, HU, AL, LA*, and *LN*
*A*	0.749	*T, RA, HU, AL, LA*, and *LN*
*He*	0.749	*T, RA, HU, AL, LA*, and *LN*

## Discussion

The AFLP technique is a reliable method, which combines the reliability of RFLPs and the power of PCR in a single method (Krumm et al. 2008) to produce highly polymorphic markers, which are used to analyse genetic diversity in plants. Garcia et al. (2004) reported that AFLP seemed to be the most appropriate molecular essay for fingerprinting and analysing genetic relationships among tropical maize inbred lines with high accuracy. It generates fingerprints of DNA from any origin or complexity (Vos et al. 1995) and produces high number of polymorphic loci. By this way, it overcomes the loss of information because of its dominance (Gerber et al. 2000) was the main reason for giving preference to AFLP method. Taking into account, the results of this study, the AFLP molecular markers could be used effectively to distinguish *I. glauca* subspecies in agreement with Gilbert et al. (2002) and Spataro et al. (2007).

When we compared the results of this study having 793 polymorphic loci, and 98.5% polymorphism, using 8 AFLP primer combinations on 67 accessions, with previous studies (Gilbert et al. 2002;Spataro et al. 2007), it was observed that this study reported a greater polymorphism among nine Turkish *Isatis glauca* subspecies populations. The genetic data analysis provides important information regarding genetic structure of the populations. This methodology also helps in screening the populations for genetic diversity within and among populations. The populations E and K displayed the higher values of genetic diversity. Population S, being a remote population to the rest of the populations, showed the least genetic diversity. The geographic distance may prevent gene transfer via pollination or other types of transfers. In addition, ecogeographical conditions in Sivas, where the population S collected are harder compared to other locations, from which the other populations collected. In addition observation of high number of unique alleles in population S displayed that ecogeograhical conditions might have effecs on the adaptation of different genotypes and causing the genetic differentiation in the population S.

AFLP fragment analysis indicated that the primer combinations E39XM40, E36XM34 and E36XM33 amplified more present fragments compared to other primer combinations. However, amplification of fragments by primer combinations varied according to populations. In general the lowest amplified present fragments were observed in population AI that had the lowest polymorphic locus and also lower genetic diversity. According to STRUCTURE analysis results, all individuals, except one are hybrid plants in population AI. This might cause the decrease in amplified fragments. During the hybridisation process, the members of the population AI might have lost some fragments. The population AI most probably inhabited in the area in the recent past according to previous field observations (personel communication with Şinasi Yıldırımlı 2013). According to fragment analysis, the highest number of present fragments were observed in population AA from Ankara/Ayaş. STRUCTURE analysis results indicated that accessions formed a distinctive and stable population structure for population AA. Although, high number of fragments were amplified, low level of genetic diversity was observed. There might be low or no gene flow with other populations due to geographic distance or some other reasons. Therefore, observed homozygosity might be higher than the other populations analysed in this study. We also considered that anthropogenic stresses might have important role on the level of genetic diversity observed in population AA. There is a paper factory in the area and also the plants generally grow on the highway road sides. These factors might cause to reduce genetic diversity in the population. On the other hand amplification of higher number of present fragments might refer that accesions from the population AA that still contain genetic upload of the original population of ssp. *galatica* in the area.

In outcrossing populations, a higher genetic variation is observed within than among populations or subpopulations (Hamrick and Godt 1990). The finding of this study showed that the gene diversity within-populations (0.76) were much larger compared to the gene diversity (0.24) between-populations is in agreement with Hamrick and Godt (1990) and Spataro et al. (2007). Although to date there is no report of detailed research about the breeding system of *I. glauca* as in the case of *I. tinctoria* (Spataro et al. 2007), these results might indicate that *I. glauca* is an outcrossing species.

The presence and frequency of unique alleles reveal useful and important information about the differentiation in genetic structure of a population. The new alleles in a population exist because of mutations and if they have ability to adapt to their environment, they have a chance to be fitted in the population gene pool. In this study, the population S had a large number (12) of unique alleles, which had frequencies higher than 0.05. The population S is geographically distant from the rest of the populations analysed in this study. Therefore, existed unique alleles in populations will drive genetic differentiation between populations as it was observed between population S and the other populations in this study.

Unique alleles have potential as favourable genes for tolerance to severe conditions, especially at higher altitudes (Mondini et al. 2010). The number and frequency of unique alleles have important features, which may be used for improvement of cultivated forms. *Isatis* L. is economically important plant especially for natural dyestuff beside its medicinal uses. The unique alleles found in *I. glauca* ssp. populations as molecular markers might be an indication of desirable traits, which can be used in the future breeding programs of *Isatis* L. plant species.

In the *Brassicaceae*, hybridization, introgression and hybrid speciation are reported as significant evolutionary forces (Marhold and Lihová 2006). Interspecific gene flow and hybridization promote evolution and species diversity of some genera, e.g. hybrids in Rorippa, as reported by Bleeker and Hurka (2001); Bleeker (2003); Schranz et al. (2005); *C. x insueta* Urbanska and *C. schulzii*, (Urbanska et al. 1997) (cited in Marhold & Lihova, 2006) and causes genetic variation (Marhold and Lihová 2006). In breeding programs, landraces or wild relatives have desirable traits such as resistance to cold, drought etc., have been used for improvement of cultivated plants (Du et al. 2009). By this way, transfer of favourable genes/gene complexes from wild allies to cultivated plant gene pools could be possible. However, in most species, incompatibility barriers between wild forms and cultivated forms usually resulted with observation of low fertility in F1 generations that constraint the possible transfer of desirable traits (Inomata, 1993;Rieseberg et al. 1995;Shivanna, 1996;Choudhary and Joshi 2001: cited in Du et al. 2009). Therefore, intertribal somatic hybrids between *Brassica napus* and *I. indigotica* (Du et al. 2009), between *Brassica rapa* and *Raphanus sativus* with dye and medicinal plant *I. indigotica* (Tu et al. 2008), and between *Brassica rapa* and *I. indigotica* (Tu et al. 2008) assessed to overcome genetic barriers by previous researchers. If intertribal hybridization is possible under control of breeders, the possibility to see natural hybrids among *Isatis* L. species or subspecies in the nature should be the expected case.

*Isatis* L. species from the *Brassicaceae*, displays morphological polymorphism and these morphological differences are often masked, even in fruits, which display the most valuable diagnostic features (Davis 1965;Hedge 1968;Jafri 1973: cited in Moazzeni et al. 2007). These variations indicate that hybridization is playing a significant role in the evolution of the genus *Isatis* L. (Moazzeni et al. 2007). The outcrossing mating system, associated with the perennial habit and vegetative reproduction is a significant factor for hybridization, constitution, and emplacement of the polyploids (Ančev 2006). Consequently, the great morphological (Görgülü et al. 2013) and genetic variation observed in subspecies of *I. glauca* in this study might be related to hybridization process. This was confirmed with the STRUCTURE analysis results, which showed that in all populations hybrid individuals were observed. Particularly, hybridisation was observed extensively in populations AG1, AG2, AI, and ANP.

According to UPGMA, PCoA and STRUCTURE analysis results, the population S was clustered as a distant population to the rest of the populations. Also, the genetic distances between population S and rest of the populations were quite high (Data not shown). This might show that formation of *I. glauca* ssp. *sivasica* is genetically differentiated obviously more than the rest of the subspecies populations analysed in this study. If the unique AFLP fragments detected in only one subspecies, were extracted, cloned, and sequenced to prepare specific primers, which might be used for simple diagnostics PCR assay of large-scale samples to identify species/subspecies or landraces (Gilbert et al. 2002).

According to the results of this study and previous studies (Yıldırımlı 1988;Görgülü et al. 2013), we suggested that Ankara/Ayaş (population AA) was the germplasm centre of *I. glauca* ssp. *galatica*. Some genotypes from Ankara/Ayaş (pop AA) migrated through Ankara/Polatlı (population ANP) and Eskişehir (population E) (Figure 2). On the other hand, some genotypes from Ankara/Ayaş (pop AA) migrated through Ankara/Gölbaşı1 (AG1), Ankara/Gölbaşı2 (AG2) and Ankara/Incek (AI) and Konya (population K). We also considered that Ankara/Beytepe was the germplasm centre of *I. glauca* ssp. *glauca* (Yıldırımlı 1988). The genotypes migrated from population AB through the area, where the populations AG1, AG2, AI, and ANP were collected. By this way ssp. *galatica* and ssp. *glauca* populations are overlaped in that area causing to a hybrid zone formation for *I. glauca* subspecies.

The populations from Eskişehir (E) and Konya (K) have distinctive population structures now and they can be recognised as the varieties of *I. glauca* ssp. *galatica*. The gene flow rates between the population ANP and the populations AG1, AG2, and AI seemed to be considerably high. Therefore, in populations AI and ANP all individuals except, one in pop AI, were hybrids.

The mutations and recombinations generates variation, which underpin genetic diversity in a population. However, selection, genetic drift and gene flow may play an important role in the genetic diversity of particularly in small size populations. The selection might be natural or artificial as it was observed in cultivated crop plant species (Suneson 1960;Frankel 1977;Nevo et al. 1984;Brown 1988;Hamrick et al. 1992: cited in Rao and Hodgkin 2002). Despite the mutations and recombinations raise the genetic diversity as evolutionary frorces in a population, the amount of molecular variation observed in this study can not be attributed to only these forces. PCA and multiple regression analysis represented the effects of environmental components. According to PCA, the environmental factors accept *LA* and *AL*, mainly contributed to the second and third components. The sum of those components is 48.55%. In addition, multiple regression analysis displayed that when all environmental factors combined they had great effect on the genetic data (*P*, *A* and *He*). These results displayed that natural selection might play important role in genetic diversity of *I. glauca* ssp. populations.

During the field studies, it was noticed that population sizes of the *I. glauca* subspecies were quite small (data not shown). Plants usually grow on the highway road sides, which negatively effect the population size because, the construction of new highways or extending road widths, remove some plant genotypes from those places or they have to move inner sides or different niches (personal communications with Şinasi Yıldırımlı 2013). Anthropogenic stressess may result in genotypes, which have desirable traits such as drought tolerance, climatic changes, etc. to be disappeared and may cause to the genetic drift or bottleneck effect in the germplasms of *I. glauca* subspecies populations.

*Isatis* L. was economically an important plant during the Roman times in both Europe and Asia (Guarino et al. 2000). Recently, an increased interest into natural dye products in textile industry raised attention to *Isatis* L. as an economically important plant once again. The leaves of *Isatis* subspecies synthesise indoxyl-forming substances, which are indican (Schunk 1855) and isatan B Beijerinck (1900), when they are exposed to the air, reflect the blue compound, indigo (Epstein et al. 1967;Gilbert et al. 2002), which is used in dying. In ancient times *I. tinctoria* L. (woad) was used for large scale indigo production in Europe. However, according to some recent phytochemical studies (Gilbert et al. 2002;Kızıl 2006;Akar 2006) *I. glauca* can also be used for indigo production. Although it was not used for large scale production, recent studies indicated that it had great potential for the production of indigo. *I. glauca* ssp. *iconia*, *I. glauca* ssp. *galatica* and *I. glauca* ssp. *sivasica* are endemic plant species of Turkey. *I. glauca* ssp. *glauc* a is native to Lebanon, Syria, Iran, and Transcaucasia (Tübives 2013;Yıldırımlı^1988^). When breeding programs for *I. glauca* are planned in future, the populations, which have high genetic diversity has more potential for breeding programs. Therefore, the genotypes, from those populations might produce high quality and amount of indigo, and have resistance to environmental stresses factors, such as fungi, bacteria, climatic, and drought, etc. should be selected for cultivation, and the promotion of large scale indigo production.

Evaluating germplasm of genetic resources is an important issue for effective conservation of plant genetic resources. Loveless and Hamrick (1984) reported that the genetic variation in plant populations is structured in space and time. What the extent of genetic diversity and its distribution in a species, it is shaped in which way are the requirements prior to determine what to conserve, and where and how to conserve it (Rao and Hodgkin 2002). Most of the molecular markers are used to determine genetic diversity and construction of genetic distances and physical map. Correlation between expression of a useful trait and a linked molecular marker could be used to construct a genetic linkage map by placing many monogenic and polygenic traits within specific regions of the plant genome (Mondini et al. 2010). Breeders may plan appropriate breeding programs for the marker assisted selection of those quality genes and introgression of these genes to develop standard varieties, which can then be used for large scale production (Gilbert et al. 2002).

## Conclusion

Compared to traditional taxonomy, which is based on morphological characters, molecular tools are used to explain taxonomy, brings in new insights into the phylogeny and taxonomy of many plant groups (Rao and Hodgkin 2002). This is the first study, in which molecular techniques were used to determine the genetic diversity and genetic differentiation among subspecies populations of *I. glauca* from Turkey.

The main conclusions of this study are as followed;

i. The results of this study showed that AFLP molecular markers could be used effectively to distinguish *I. glauca* subspecies.
ii. According to molecular data, the UPGMA, PCoA and STRUCTURE analysis, results showed that the accessions from the population S was clustered as a remote population to the rest of the populations. Populations AA, AB, E, K and S had distinctive population structures.
iii. It is suggested that the population AA is the origin of subspecies *I. glauca* ssp. *galatica* germplasm center, while the population AB was the origin of subspecies *I. glauca* ssp. *glauca*. germplasm center.
iv. The populations from Eskişehir (E) and Konya (K) were recognised as the varieties of *I. glauca* ssp. *galatica*.
v. Ankara province might be the area, where species *Isatis glauca* Aucher ex Boiss. originated.
vi. The genetic information obtained in the present study will assist to acquire more information about the population genetic structure, the basis for speciation or subspecies formation in *Isatis glauca* species and its taxonomy. Consequently, the subspecies and their varieties will be allocated taxonomically in appropriate way.

## Authors' information

EG is doing her PH.D at Hacettepe University in Ankara, Turkey.

ÖÖ is an assistant professor at Hitit University, Faculty of Art and Science, Department of Biology, Çorum, Turkey.

ŞY is a professor at Hacettepe University, Faculty of Science, and Department of Biology in Ankara, Turkey.

## Electronic supplementary material

Additional file 1: Table S1: Accession numbers (AN), population codes (PC), the province, where the accessions were collected and subspecies name of the accessions analysed in this study. Accession numbers were given by Professor Dr. Şinasi Yıldırımlı. First two letters represent the population code, which the accession belongs to it. The first number represent collection year and the second number(s) represents the accession number assigned. (DOCX 25 KB)

Additional file 2: Table S2: The primers and adaptors used in this study (Abbreviations: Primer code PR.C, *Eco* R I E, and *Mse* I M). (DOCX 19 KB)

Additional file 3: Table S3: Primer combinations used in this study; *Eco* R I primers, labelled with FAM and VIC florescent dye and *Mse* I primers unlabelled (Abbreviations: Fluorescent label FL, *Eco* R I E, and *Mse* I M). (DOCX 18 KB)

Additional file 4: Table S4: Selective PCR amplification program, used in this study. (DOCX 18 KB)

Additional file 5: Table S5: Climatic (Temperature T, Humidity HU, and Rainfall RA) data of the places, where the populations were collected (Population code PC). (DOCX 19 KB)

Additional file 6: Table S6: Private alleles observed in studied nine Turkish *Isatis glauca* subspecies populations. (DOCX 19 KB)

Additional file 7: Table S7: Component matrix of variables and their contributions to principal components (Abbreviations: Number of polymorphic locus *P*, average number of allele *A*, average number of allele per polymorphic locus *A* P, genetic diversity *He*, temperature *T*, rainfall *RA*, humidity *HU*, altitude *AL*, latitude *LA* and longitude *LN*). (DOCX 20 KB)

Below are the links to the authors’ original submitted files for images.

Authors’ original file for figure 1

Authors’ original file for figure 2

Authors’ original file for figure 3

Authors’ original file for figure 4

Authors’ original file for figure 5

Authors’ original file for figure 6

Authors’ original file for figure 7
