# Supplementary material for: Genetic diversity in populations of Isatis glauca Aucher ex Boiss. ssp. from Central Anatolia in Turkey, as revealed by AFLP analysis
Source: Bot Stud. 2013 Nov 4;54:48. doi: 10.1186/1999-3110-54-48 (PMC5430366; doi:10.1186/1999-3110-54-48)
Supplement: Supplementary file 1 — Additional file 1: Table S1: Accession numbers (AN), population codes (PC), the province, where the accessions were collected and subspecies name of the accessions analysed in this study. Accession numbers were given by Professor Dr. Şinasi Yıldırımlı. First two letters represent the population code, which the accession belongs to it. The first number represent collection year and the second number(s) represents the accession number assigned. (DOCX 25 KB) [file 40529_2013_98_MOESM1_ESM.docx]

**ADDITIONAL FILE 1**

**Table S1** Accession numbers (AN), population codes (PC), the province, where the accession were collected and subspecies name of the accessions analysed in this study. Accession numbers were given by Professor Dr. Şinasi Yıldırımlı. First two letters represent the population code, which the accession belongs to it. The first number represent collection year and the second number(s) represents the accession number assigned.

| N | AN | PC | Province | Subspecies | N | AN | PC | Province | Subspecies | N | AN | PC | Province | Subspecies |
| --- | --- | --- | --- | --- | --- | --- | --- | --- | --- | --- | --- | --- | --- | --- |
| 1. | AA/11-1 | AA | Ankara/Ayas | *galatica* | 24. | AG1/11-9 | AG1 | Ankara/Golbası | *glauca* | 47. | E/11-3 | E | Eskisehir | *galatica* |
| 2. | AA/11-4 | AA | Ankara/Ayas | *galatica* | 25. | AG2/11-1 | AG2 | Ankara/Golbası | *galatica* | 48. | E/11-4 | E | Eskisehir | *galatica* |
| 3. | AA/11-5 | AA | Ankara/Ayas | *galatica* | 26. | AG2/11-2 | AG2 | Ankara/Golbası | *galatica* | 49. | E/11-9 | E | Eskisehir | *galatica* |
| 4. | AA/11-6 | AA | Ankara/Ayas | *galatica* | 27. | AG2/11-3 | AG2 | Ankara/Golbası | *galatica* | 50. | E/11-10 | E | Eskisehir | *galatica* |
| 5. | AA/11-7 | AA | Ankara/Ayas | *galatica* | 28. | AG2/11-8 | AG2 | Ankara/Golbası | *galatica* | 51. | K/11-2 | K | Konya | *galatica* |
| 6. | AA/11-10 | AA | Ankara/Ayas | *galatica* | 29. | AG2/11-9 | AG2 | Ankara/Golbası | *galatica* | 52. | K/11-1 | K | Konya | *galatica* |
| 7. | AA/11-11 | AA | Ankara/Ayas | *galatica* | 30. | AG2/11-10 | AG2 | Ankara/Golbası | *galatica* | 53. | K/11-4 | K | Konya | *galatica* |
| 8. | AA/11-12 | AA | Ankara/Ayas | *galatica* | 31. | AG2/11-11 | AG2 | Ankara/Golbası | *galatica* | 54. | K/11-6 | K | Konya | *galatica* |
| 9. | AB/11-1 | AB | Ankara/Beytepe | *glauca* | 32. | AG2/11-13 | AG2 | Ankara/Golbası | *galatica* | 55. | K/11-7 | K | Konya | *galatica* |
| 10. | AB/11-2 | AB | Ankara/Beytepe | *glauca* | 33. | AI/11-1 | AI | Ankara/Beytepe | *glauca* | 56. | K/11-8 | K | Konya | *galatica* |
| 11. | AB/11-4 | AB | Ankara/Beytepe | *glauca* | 34. | AI/11-2 | AI | Ankara/Incek | *glauca* | 57. | K/11-9 | K | Konya | *galatica* |
| 12. | AB/11-6 | AB | Ankara/Beytepe | *glauca* | 35. | AI/11-3 | AI | Ankara/Incek | *glauca* | 58. | K/11-10 | K | Konya | *galatica* |
| 13. | AB/11-7 | AB | Ankara/Beytepe | *glauca* | 36. | AI/11-5 | AI | Ankara/Incek | *glauca* | 59. | K/11-11 | K | Konya | *galatica* |
| 14. | AB/11-9 | AB | Ankara/Beytepe | *glauca* | 37. | AI/11-7 | AI | Ankara/Incek | *glauca* | 60. | S/11-2 | S | Sivas | *sivasica* |
| 15. | AB/11-10 | AB | Ankara/Beytepe | *glauca* | 38 | AI/11-12 | AI | Ankara/Incek | *glauca* | 61. | S/11-3 | S | Sivas | *sivasica* |
| 16. | AB/11-12 | AB | Ankara/Beytepe | *glauca* | 39 | ANP/11-5 | ANP | Ankara/Polatlı | *galatica* | 62. | S/11-4 | S | Sivas | *sivasica* |
| 17. | AG1/11-1 | AG1 | Ankara/Golbası | *glauca* | 40. | ANP/11-6 | ANP | Ankara/Polatlı | *galatica* | 63. | S/11-5 | S | Sivas | *sivasica* |
| 18. | AG1/11-2 | AG1 | Ankara/Golbası | *glauca* | 41. | ANP/11-7 | ANP | Ankara/Polatlı | *galatica* | 64. | S/11-6 | S | Sivas | *sivasica* |
| 19. | AG1/11-3 | AG1 | Ankara/Golbası | *glauca* | 42. | ANP/11-21 | ANP | Ankara/Polatlı | *galatica* | 65. | S/11-7 | S | Sivas | *sivasica* |
| 20. | AG1/11-4 | AG1 | Ankara/Golbası | *glauca* | 43. | ANP/11-33 | ANP | Ankara/Polatlı | *galatica* | 66. | S/11-8 | S | Sivas | *sivasica* |
| 21. | AG1/11-6 | AG1 | Ankara/Golbası | *glauca* | 44. | ANP/11-38 | ANP | Ankara/Polatlı | *galatica* | 67 | S/11-9 | S | Sivas | *sivasica* |
| 22. | AG1/11-7 | AG1 | Ankara/Golbası | *glauca* | 45 | ANP/11-51 | ANP | Ankara/Polatlı | *galatica* |  |  |  |  |  |
| 23. | AG1/11-8 | AG1 | Ankara/Golbası | *glauca* | 46 | E/11-2 | E | Eskisehir | *galatica* |  |  |  |  |  |
