# Supplementary material for: Genetic diversity in populations of Isatis glauca Aucher ex Boiss. ssp. from Central Anatolia in Turkey, as revealed by AFLP analysis
Source: Bot Stud. 2013 Nov 4;54:48. doi: 10.1186/1999-3110-54-48 (PMC5430366; doi:10.1186/1999-3110-54-48)
Supplement: Supplementary file 2 — Additional file 2: Table S2: The primers and adaptors used in this study (Abbreviations: Primer code PR.C, Eco R I E, and Mse I M). (DOCX 19 KB) [file 40529_2013_98_MOESM2_ESM.docx]

**ADDITIONAL FILE 2**

**Table S2.** The primers and adaptors used in this study (Abbreviations: Primer code PR.C, *Eco*R I E, and *Mse* I M)

|  | PR.C | Primer/adaptor name | Sequences (5’-3’) |
| --- | --- | --- | --- |
| Adaptors |  | *Eco*RI adaptör-1 | 5’-CTC GTA GAC TGC GTA CC-3’ |
|  |  |  | 3’-CAT CTG ACG CAT GG-5’ |
|  |  | *Mse*I adaptör-1 | 5’-GAC GAT GAG TCC TGA G-3’ |
|  |  |  | 3’-TA CTC AGG ACT C-5’ |
| Preselective primers | (E01) | *Eco*RI+A | 5’-GAC TGC GTA CCA ATT CA-3’ |
|  | (M01) | *Mse*I+A | 5’-GAT GAG TCC TGA GTA AA-3’ |
| Selective primers | (E33) | *EcoRI +*AAG | 5’-GAC TGC GTA CCA ATT CAA G-3’ |
|  | (E36) | *Eco*RI+ACC | 5’-GAC TGC GTA CCA ATT CAC C-3’ |
|  | (E39) | *EcoR*I*+*AGA | 5’-GAC TGC GTA CCA ATT CAG A-3’ |
|  | (M32) | *Mse*I+AAC | 5’-GAT GAG TCC TGA GTA AC-3’ |
|  | (M33) | *Mse*I*+*AAG | 5’-GAT GAG TCC TGA GTA AG-3’ |
|  | (M34) | *Mse*I*+*AAT | 5’-GAT GAG TCC TGA GTA AT-3’ |
|  | (M35) | *Mse*I*+*ACA | 5’-GAT GAG TCC TGA GTA CA-3’ |
|  | (M38) | *Mse*I*+*ACT | 5’-GAT GAG TCC TGA GTA CT-3’ |
|  | (M40) | *Mse*I*+*AGC | 5’-GAT GAG TCC TGA GTA GC-3’ |
|  | (M41) | *Mse*I*+*AGG | 5’-GAT GAG TCC TGA GTA GG-3’ |
