# Supplementary material for: Genetic diversity in populations of Isatis glauca Aucher ex Boiss. ssp. from Central Anatolia in Turkey, as revealed by AFLP analysis
Source: Bot Stud. 2013 Nov 4;54:48. doi: 10.1186/1999-3110-54-48 (PMC5430366; doi:10.1186/1999-3110-54-48)
Supplement: Supplementary file 3 — Additional file 3: Table S3: Primer combinations used in this study; Eco R I primers, labelled with FAM and VIC florescent dye and Mse I primers unlabelled (Abbreviations: Fluorescent label FL, Eco R I E, and Mse I M). (DOCX 18 KB) [file 40529_2013_98_MOESM3_ESM.docx]

**ADDITIONAL FILE 3**

**Table S3.** Primer combinations used in this study; *Eco*R I primers, labelled with FAM and VIC florescent dye and *Mse* I primers unlabelled (Abbreviations: Fluorescent label FL, *Eco*R I E, and *Mse* I M)

|  | FL | Primer combinations |
| --- | --- | --- |
|  | FAM -1 | E39-M40 |
|  | FAM -2 | E36-M34 |
|  | FAM-3 | E39-M41 |
|  | FAM-4 | E36-M32 |
|  | VIC-1 | E33-M38 |
|  | VIC-2 | E36-M33 |
|  | VIC-3 | E33-M40 |
|  | VIC-4 | E36-M35 |
