# Supplementary material for: Genetic diversity in populations of Isatis glauca Aucher ex Boiss. ssp. from Central Anatolia in Turkey, as revealed by AFLP analysis
Source: Bot Stud. 2013 Nov 4;54:48. doi: 10.1186/1999-3110-54-48 (PMC5430366; doi:10.1186/1999-3110-54-48)
Supplement: Supplementary file 4 — Additional file 4: Table S4: Selective PCR amplification program, used in this study. (DOCX 18 KB) [file 40529_2013_98_MOESM4_ESM.docx]

**ADDITIONAL FILE 4**

**Table S4.** Selective PCR amplification program, used in this study

|  | PCR steps | T | Time | Cycles |
| --- | --- | --- | --- | --- |
| 1 | Extended initial denaturation | 94 °C | 2 min |  |
| 2 | Denaturation | 94 °C | 45sec |  |
| 3 | Annealing | 66 °C | 45 sec | Reducing each step 1 °C till 56 °C |
| 4 | Extension | 72 °C | 2 min | 10 times, go to 2. step |
| 5 | Denaturation | 94 °C | 45 sec |  |
| 6 | Annealing | 56 °C | 45 sec |  |
| 7 | Extension | 72 °C | 2 min | 10 times go to 5. step |
| 8 | Final extension | 60 °C | 30 min |  |
| 9 | ∞ | 4 °C | - |  |
| 10 | End |  |  |  |
