# Supplementary material for: Genetic diversity in populations of Isatis glauca Aucher ex Boiss. ssp. from Central Anatolia in Turkey, as revealed by AFLP analysis
Source: Bot Stud. 2013 Nov 4;54:48. doi: 10.1186/1999-3110-54-48 (PMC5430366; doi:10.1186/1999-3110-54-48)
Supplement: Supplementary file 5 — Additional file 5: Table S5: Climatic (Temperature T, Humidity HU, and Rainfall RA) data of the places, where the populations were collected (Population code PC). (DOCX 19 KB) [file 40529_2013_98_MOESM5_ESM.docx]

**ADDITIONAL FILE 5**

**Table S5.** Climatic (Temperature T, Humidity HU, and Rainfall RA) data of the places, where the populations were collected (Population code PC)

| PC | Year | T (°C) | HU (%) | RA (mm) |
| --- | --- | --- | --- | --- |
| AA | 2011 | 23.65 | 61.58 | 34.11 |
| AB | 2011 | 23.65 | 61.58 | 34.11 |
| AG1 | 2011 | 21.61 | 63.61 | 36.37 |
| AG2 | 2011 | 21.61 | 63.61 | 36.37 |
| AI | 2011 | 23.65 | 61.58 | 36.37 |
| ANP | 2011 | 23.68 | 62.79 | 30.99 |
| E | 2011 | 23.77 | 62.68 | 30.14 |
| K | 2011 | 24.38 | 44.8 | 34.45 |
| S | 2011 | 22.63 | 61.91 | 40.14 |
