# Supplementary material for: Genetic diversity in populations of Isatis glauca Aucher ex Boiss. ssp. from Central Anatolia in Turkey, as revealed by AFLP analysis
Source: Bot Stud. 2013 Nov 4;54:48. doi: 10.1186/1999-3110-54-48 (PMC5430366; doi:10.1186/1999-3110-54-48)
Supplement: Supplementary file 6 — Additional file 6: Table S6: Private alleles observed in studied nine Turkish Isatis glauca subspecies populations. (DOCX 19 KB) [file 40529_2013_98_MOESM6_ESM.docx]

**ADDITIONAL FILE 6**

**Table S6** Private alleles observed in studied nine Turkish *Isatis glauca* subspecies populations

| Locus Name | Allele | Frequency | Found in Population |
| --- | --- | --- | --- |
| E36/M32-157 | 1 | 1.00 | S |
| E36/M32-309 | 1 | 0.71 | S |
| E36/M34-188 | 1 | 0.86 | S |
| E36/M34-281 | 1 | 0.86 | S |
| E36/M32-58 | 4 | 0.13 | AG1 |
| E36/M32-83 | 1 | 0.63 | S |
| E36/M32-101 | 1 | 0.63 | S |
| E36/M32-201 | 1 | 0.63 | AA |
| E33/M38-181 | 0 | 0.75 | S |
| E33/M38-183 | 0 | 0.38 | S |
| E33/M38- 242 | 0 | 0.13 | S |
| E33/M38-341 | 0 | 0.20 | E |
| E33/M38-420 | 1 | 0.75 | AA |
| E33/M38-425 | 1 | 0.63 | S |
| E36/M33-110 | 0 | 0.13 | AG 2 |
| E36/M33-137 | 0 | 0.13 | AG 2 |
| E36/M33-140 | 1 | 1.00 | S |
| E33/M40-118 | 1 | 0.71 | S |
| E36/M35-117 | 0 | 0.29 | AG 2 |
