# Supplementary material for: Genetic diversity in populations of Isatis glauca Aucher ex Boiss. ssp. from Central Anatolia in Turkey, as revealed by AFLP analysis
Source: Bot Stud. 2013 Nov 4;54:48. doi: 10.1186/1999-3110-54-48 (PMC5430366; doi:10.1186/1999-3110-54-48)
Supplement: Supplementary file 7 — Additional file 7: Table S7: Component matrix of variables and their contributions to principal components (Abbreviations: Number of polymorphic locus P, average number of allele A, average number of allele per polymorphic locus A P, genetic diversity He, temperature T, rainfall RA, humidity HU, altitude AL, latitude LA and longitude LN). (DOCX 20 KB) [file 40529_2013_98_MOESM7_ESM.docx]

**ADDITIONAL FILE 7**

**Table S7.** Component matrix of variables and their contributions to principal components (Abbreviations: Number of polymorphic locus *P*, average number of allele *A*, average number of allele per polymorphic locus *A*_P_, genetic diversity *He*, temperature *T*, rainfall *RA*, humidity *HU*, altitude *AL*, latitude *LA* and longitude *LN*)

|  |  | Components |  |
| --- | --- | --- | --- |
| Variables | 1 | 2 | 3 |
| *N* | 0.24 | **0.87** | 0.11 |
| *P* | **0.86** | 0.49 | -0.03 |
| *A* | **0.86** | 0.49 | -0.03 |
| *He* | **0.81** | -0.15 | -0.30 |
| *AL* | **-0.77** | 0.52 | -0.01 |
| *LT* | -0.38 | -0.26 | **0.67** |
| *LN* | -0.62 | **0.59** | 0.22 |
| *T* | 0.47 | -0.27 | **0.80** |
| *HU* | -0.29 | -0.40 | **-0.79** |
| *RA* | -0.29 | **0.89** | -0.18 |

*Highlighted numbers indicate contribution of variables to the corresponded principal components.
