# Supplementary figures and images for: Genetic diversity in populations of Isatis glauca Aucher ex Boiss. ssp. from Central Anatolia in Turkey, as revealed by AFLP analysis
Source: Bot Stud. 2013 Nov 4;54:48. doi: 10.1186/1999-3110-54-48 (PMC5430366; doi:10.1186/1999-3110-54-48)

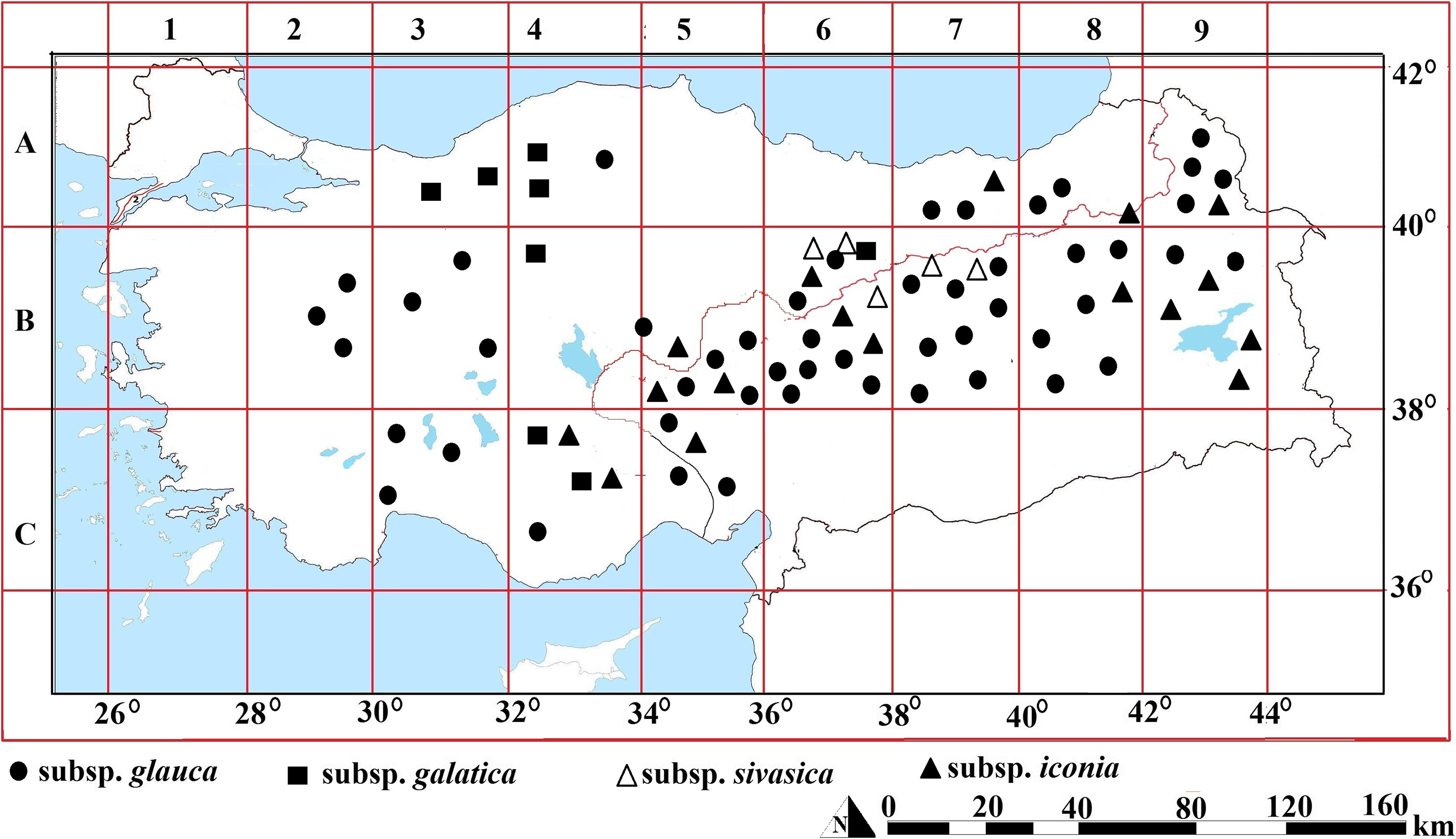

Supplement: Supplementary file 8 — Authors’ original file for figure 1 [file 40529_2013_98_MOESM8_ESM.tif]

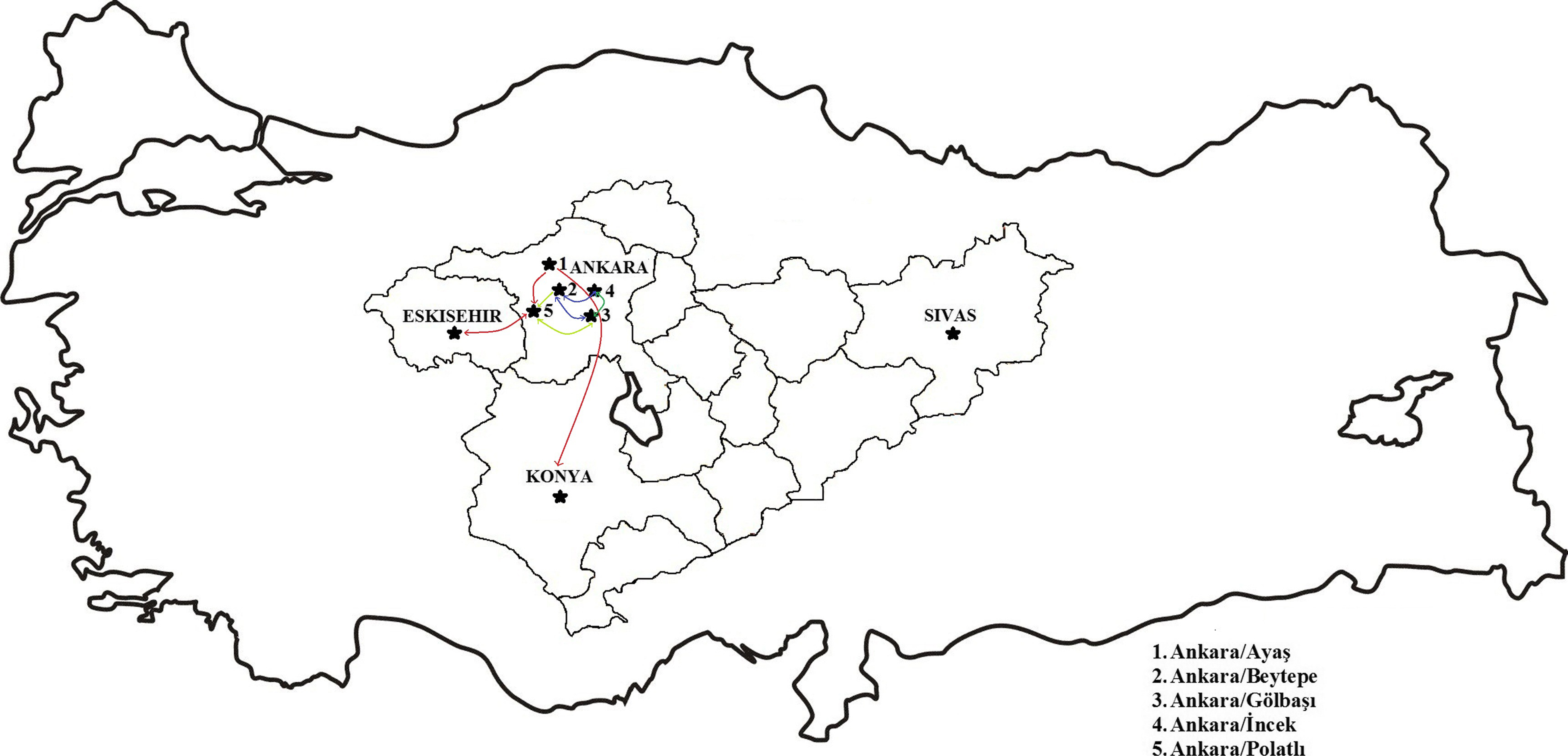

Supplement: Supplementary file 9 — Authors’ original file for figure 2 [file 40529_2013_98_MOESM9_ESM.tif]

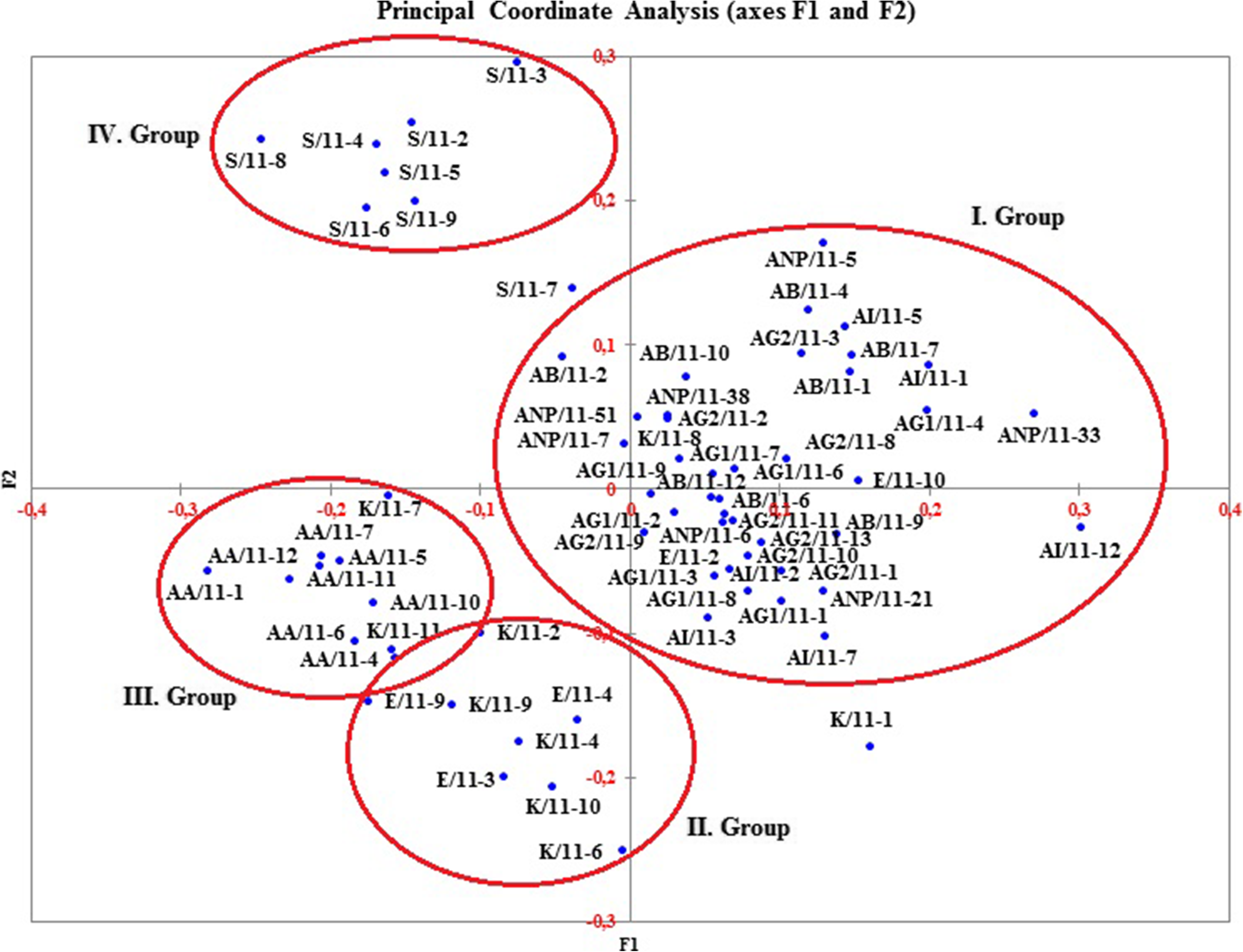

Supplement: Supplementary file 10 — Authors’ original file for figure 3 [file 40529_2013_98_MOESM10_ESM.tif]

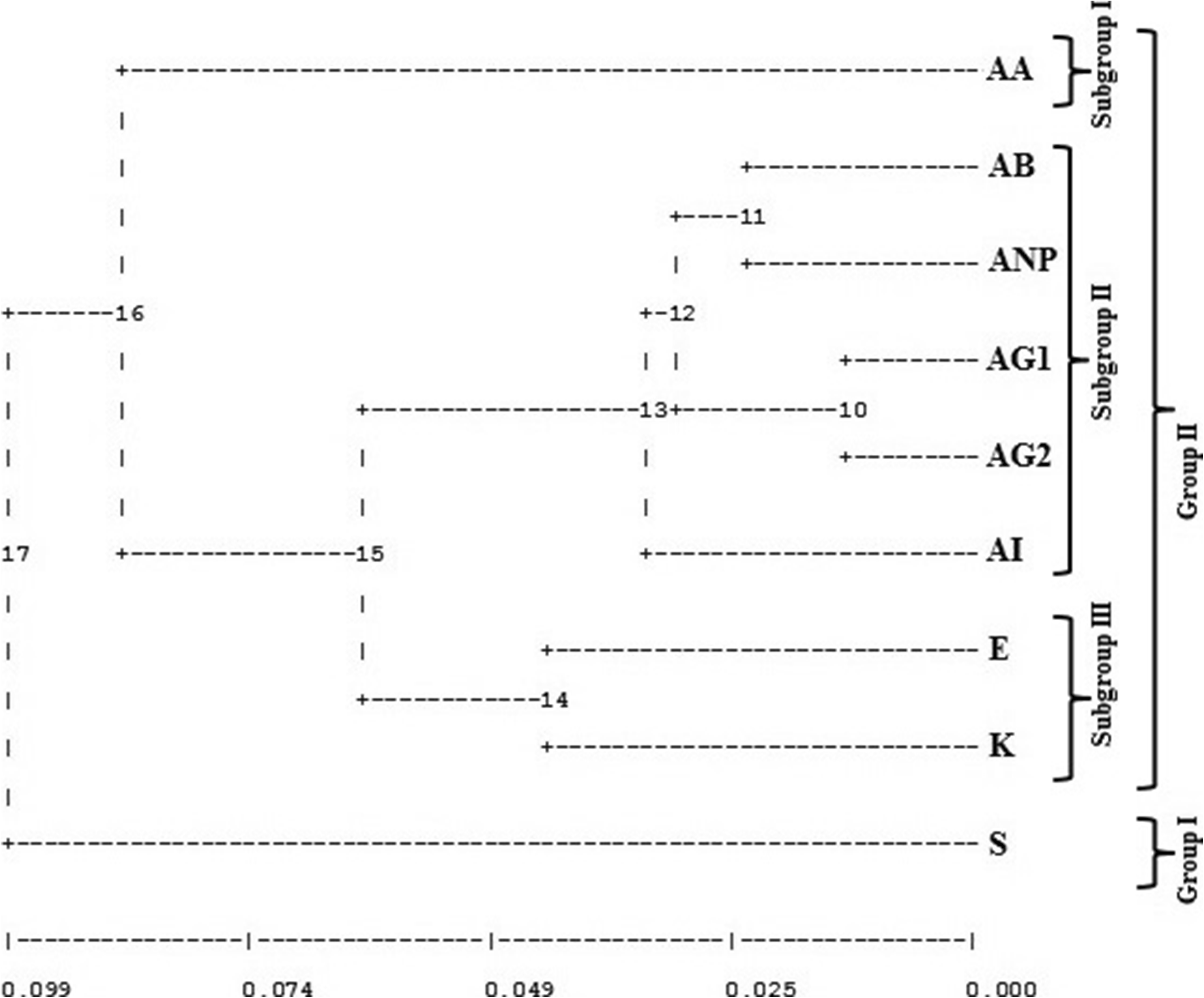

Supplement: Supplementary file 11 — Authors’ original file for figure 4 [file 40529_2013_98_MOESM11_ESM.tif]

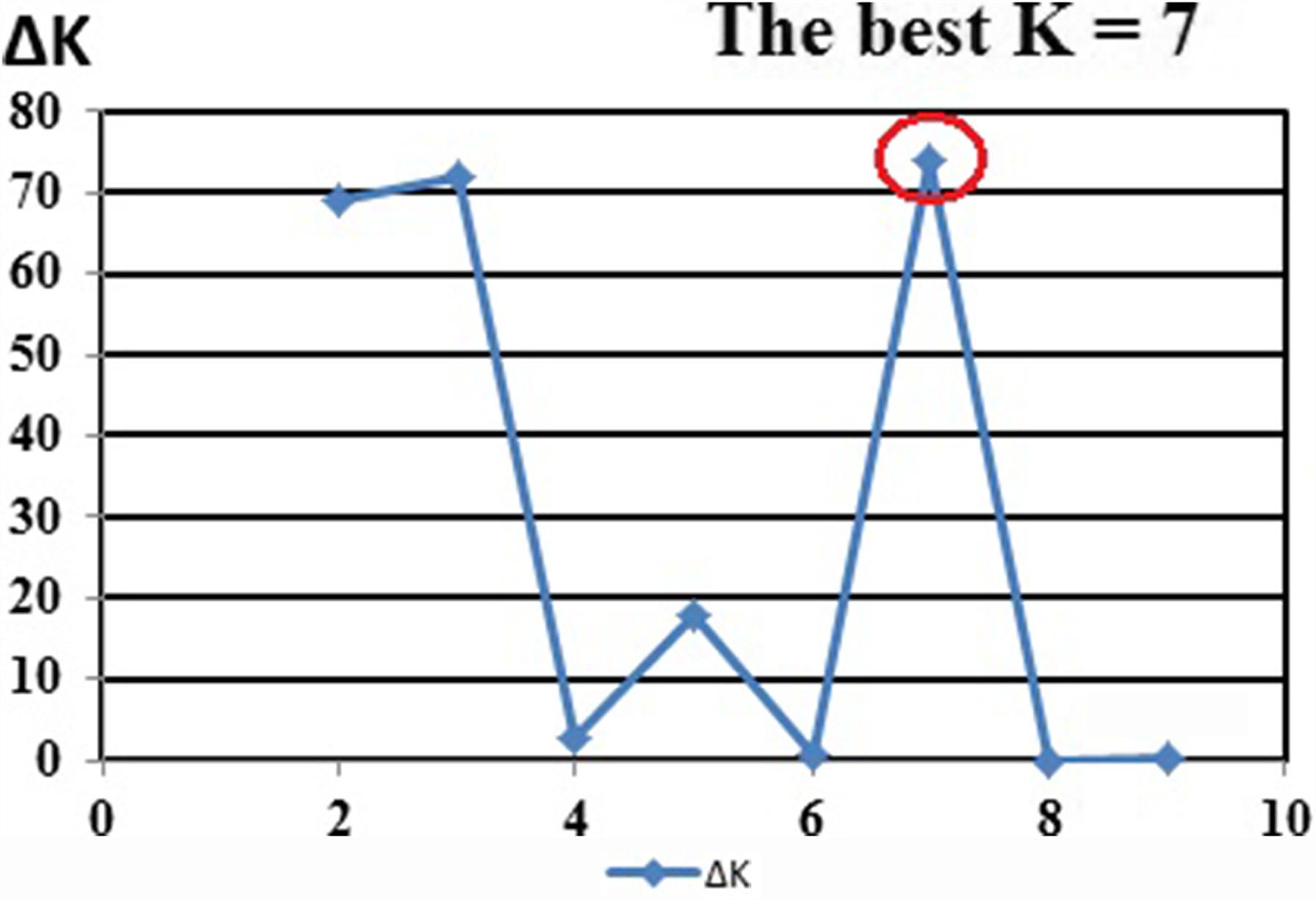

Supplement: Supplementary file 12 — Authors’ original file for figure 5 [file 40529_2013_98_MOESM12_ESM.tif]

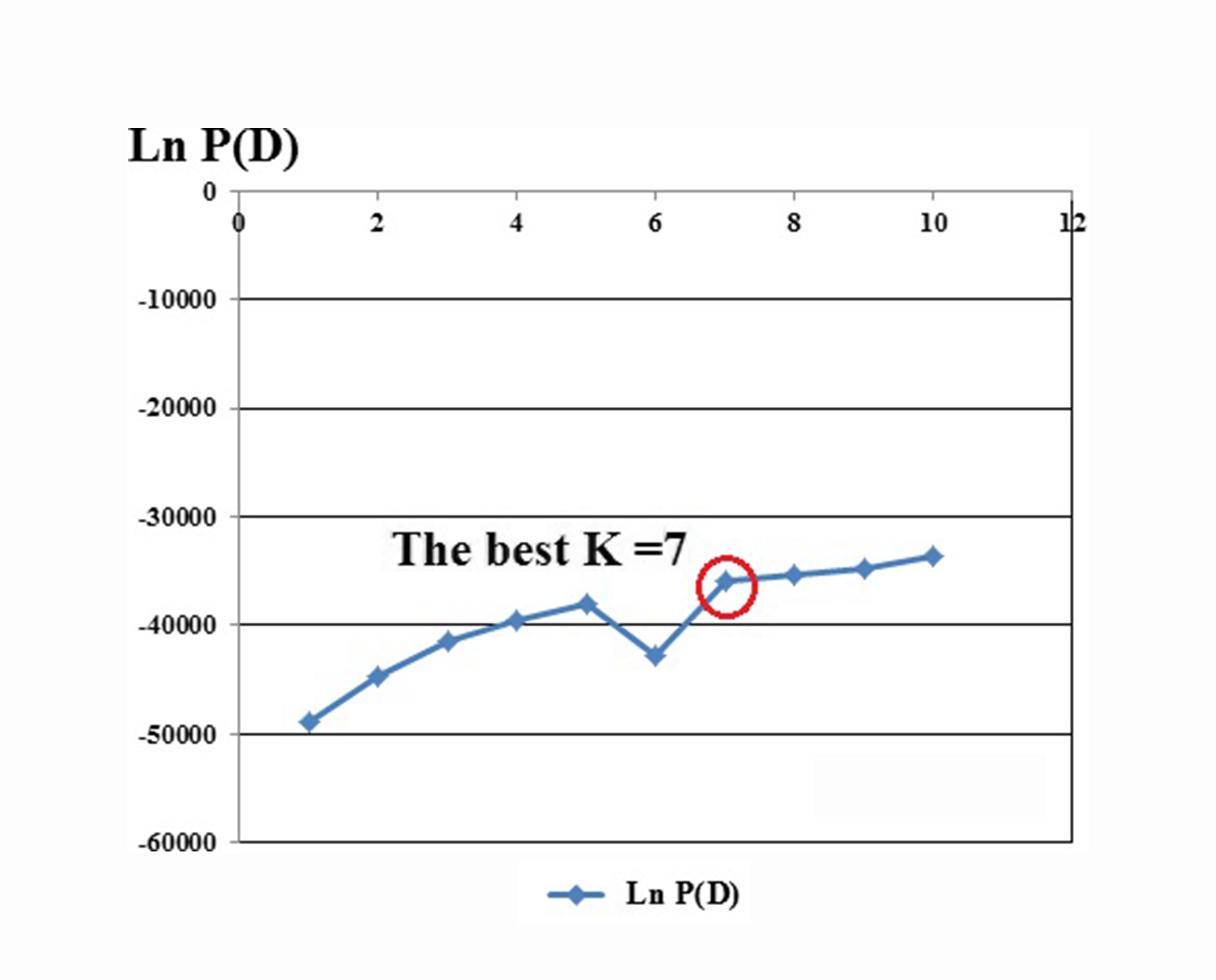

Supplement: Supplementary file 13 — Authors’ original file for figure 6 [file 40529_2013_98_MOESM13_ESM.tif]

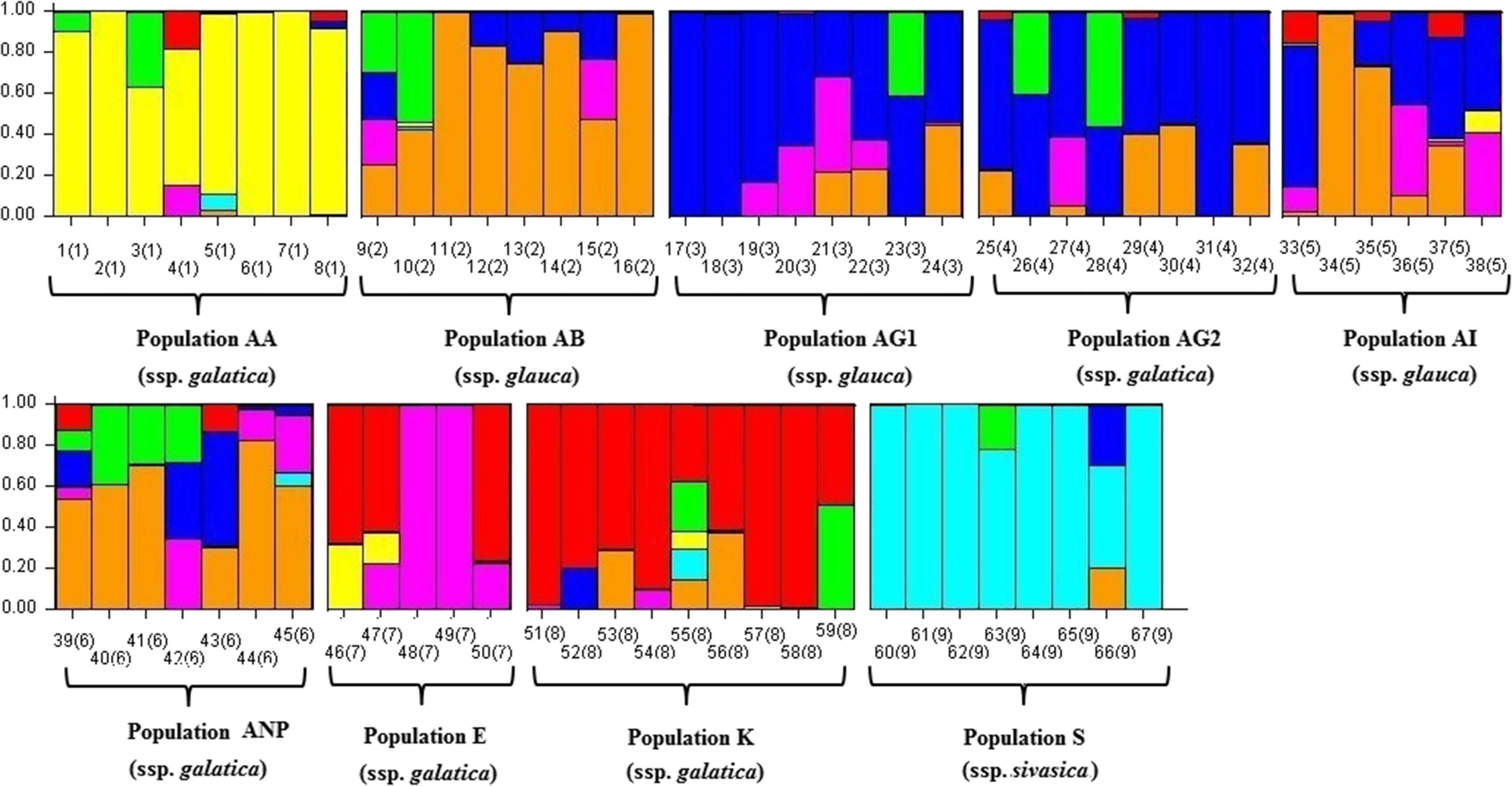

Supplement: Supplementary file 14 — Authors’ original file for figure 7 [file 40529_2013_98_MOESM14_ESM.tif]
